# Descriptions of a new species of genus *Angarotipula* and life history of *Angarotipulalaetipennis* (Alexander, 1935) (Diptera, Tipulidae)

**DOI:** 10.3897/BDJ.10.e82427

**Published:** 2022-06-10

**Authors:** Qi-Cheng Yang, Bing Zhang, Ding Yang, Xiaoyan Liu

**Affiliations:** 1 Huazhong Agricultural University, Wuhan, China Huazhong Agricultural University Wuhan China; 2 China Agricultural University, Beijing, China China Agricultural University Beijing China

**Keywords:** crane flies, egg, feeding habits, larva, pupa, morphology, taxonomy

## Abstract

**Background:**

*Angarotipula* is a small genus of Tipulidae with only fourteen described species in the world and seven known species in China.

**New information:**

Here, one new species is added to the fauna of Sichuan. The life history of *Angarotipulalaetipennis* (Alexander, 1935) is presented and the morphologies of all stages are described.

## Introduction

*Angarotipula* is a small genus of Tipulidae with only fourteen described species in the world (Oosterbroek 2022). This genus is characterised by the following features: body middle-sized, head and thorax generally greyish-brown or black, tergites usually with black central stripe, wings tinted yellowish-brown without other markings; nasus distinct, flagellomeres lacking verticiles, broadened near middle of flagellomere, tibial spur formula 1-1-2, small genitalia in both sexes, outer gonostylus rod-like or subtriangular, inner gonostylus usually with long and obtuse beak (Anonymous 1961, Brodo 2017, Liu and Yang 2010). It is distributed in Asia and North America, of which seven species are distributed in China, four species have been known to occur in India, two species are known from North America, two species from Russia and Mongolia and one species from Laos (Oosterbroek 2022).

In this paper, one species, *A.tristis*, sp. n. from Sichuan, China is described as new to science. The detailed descripitons and figures of eggs, larvae and pupae of *A.laetipennis* (Alexander, 1935) are offered and the life history of this species is reported. The feeding habits of genus *Angarotipula* are reported for first time.

## Materials and methods

**Rearing of larvae**

Male and female adults were collected from Luxi County, Yunnan Province and placed in a plastic water bottle to mate. The eggs were laid in shallow water. After hatching, the larvae were transferred to a foam box with attachments like wooden sticks and coontail (*Ceratophyllumdemersum*) and fed algae feed (*Chlorellavulgaris*). Sand and gravel were used to make slopes for pupariation of mature larvae. All larvae were reared in Luxi County under natural conditions (T = 22-32°C, H = 40-60%).

The spiracular disc of the 1st instar larva is drawn under an OLYMPUS BX43 microscope. All other specimens were studied and illustrated with OLYMPUS SZ61 stereomicroscope. Details of colouration were checked in specimens immersed in 75% ethyl alcohol (C_2_H_5_OH), to observe pruinescence after drying. Genitalic preparations of males and head shell of last instar larvae were made using Lactic acid solution (C_3_H_6_O_3_ > 85%) heated in a water-bath to 95-97°C for 4-6 minutes and cooled down to room temperature. After examination, it was transferred to fresh glycerine (C_3_H_8_O_3_) and stored in a microvial tied to the specimen.

Type specimens are deposited in the Entomological Museum of China Agricultural University (CAU), Beijing. The distribution map was obtained from Arcmap and modified with Adobe Photoshop CS6. All pictures were adjusted and assembled into plates with Adobe Photoshop CS6.

The morphological terminology of the male hypopygium mostly follows that of Cumming and Wood (2017) and de Jong (2017). The terminology for describing the larvae and pupae follows Gelhaus (1986), Young (2004), Podeniene et al. (2006) and Neugart et al. (2009). Arrangement of setae of last instar larva labelling was according to Gelhaus (1986), Podeniene et al. (2006) and Brodo (2017). The following abbreviations are used in figures: ig = inner gonostylus, og = outer gonostylus, st VIII = sternite eight, st IX = sternite nine, tg VIII = tergite eight, tg IX = tergite nine, bk = beak.

## Taxon treatments

### Angarotipula tristis

Yang, 2022 sp. n.

439DD83D-E56E-53E0-AEC9-235492F5330A

C268B3B5-96EA-4CB7-971B-95EC8A86662E

#### Materials

- **Type status:** Holotype. **Occurrence:** recordedBy: Cai Xiao-Dong; sex: male; **Taxon:** higherClassification: Animalia; Arthropoda; Insecta; Diptera; Nematocera; Tipulomorpha; Tipuloidea; Tipulidae; Tipulinae; Angarotipula; kingdom: Animalia; phylum: Arthropoda; class: Insecta; order: Diptera; family: Tipulidae; genus: Angarotipula; specificEpithet: *tristis*; **Location:** continent: Asia; country: China; countryCode: CN; stateProvince: Sichuan; county: Ganzi Tibetan Autonomous Prefecture; municipality: Daocheng; locality: Yading Hongcaodi (亚丁 红草地); verbatimLocality: Hongcaodi (红草地); verbatimElevation: 3751 m; verbatimLatitude: 29°06'11"N; verbatimLongitude: 100°13'59.6"E; **Identification:** identifiedBy: Yang Qi-Cheng; **Event:** year: 2019; month: 7; day: 9; verbatimEventDate: 9-VII-2019; habitat: intermittent wetlands; fieldNotes: Plant dominant species: Polygonaceae; **Record Level:** type: PhysicalObject; language: en; institutionCode: CAU
- **Type status:** Paratype. **Occurrence:** recordedBy: Cai Xiao-Dong; sex: 2 males; **Taxon:** higherClassification: Animalia; Arthropoda; Insecta; Diptera; Nematocera; Tipulomorpha; Tipuloidea; Tipulidae; Tipulinae; Angarotipula; kingdom: Animalia; phylum: Arthropoda; class: Insecta; order: Diptera; family: Tipulidae; genus: Angarotipula; specificEpithet: *tristis*; **Location:** continent: Asia; country: China; countryCode: CN; stateProvince: Sichuan; county: Ganzi Tibetan Autonomous Prefecture; municipality: Daocheng; locality: Yading Hongcaodi (亚丁 红草地); verbatimLocality: Hongcaodi (红草地); verbatimElevation: 3751 m; verbatimLatitude: 29°06'11"N; verbatimLongitude: 100°13'59.6"E; **Identification:** identifiedBy: Yang Qi-Cheng; **Event:** year: 2019; month: 7; day: 9; verbatimEventDate: 9-VII-2019; habitat: intermittent wetlands; fieldNotes: Plant dominant species: Polygonaceae; **Record Level:** type: PhysicalObject; language: en; institutionCode: CAU

#### Description

Males (n = 3): body length 11.3–11.5 mm, wing length 13.4–13.6 mm, antenna length 2.9–3.0 mm.

**Figure 1.**
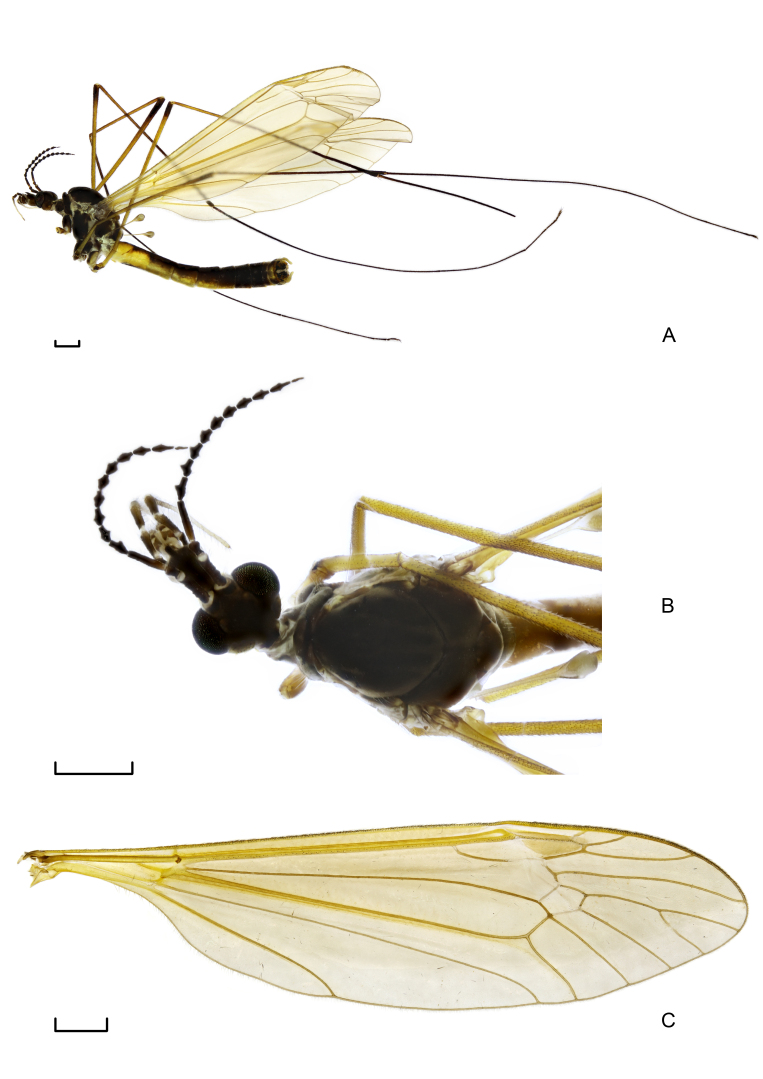
*Angarotipulatristis*, sp. n., male holotype. (A) Habitus, lateral view; (B) Wing; (C) Habitus, dorsal view. Scale bars = 1.0 mm.

Head (Fig. 1A, B). Mainly brownish-black. Occiput without marking. Rostrum dark brown, except black dorsally. Nasus black. Gena yellowish-white. Setae on head pale yellow. Head with greyish-yellow pruinescence. Antennal scape brownish-black, with a white membranous and nearly round notch at apex. Pedicel dark brown. First flagellomere cylindrical, 1.25 times as long as scape, black with brown base with short thin black setae. Other flagellomeres black, broadened near middle, with few short black setae and appressed pale pubescence. Labellum and palpus brownish-black, except distal segment of palpus yellowish-brown, labellum and palpus with black setae.

Thorax (Fig. 1A and B). Mainly black. Pronotum, prescutum, scutum and mediotergite black. Scutellum brownish-black. Pleuron black, with greyish-yellow pruinescence. Setae on dorsum of thorax pale yellow. Legs with first and second coxae black, third coxae with anterior 1/3 black and posterior 2/3 yellow, femora dark yellow with black basal margins and dark brown apexes; tibiae yellowish-brown, tarsi dark brown to black. Setae on legs brownish-black. Wing pale yellowish, areas surrounding major longitudinal veins strikingly yellowish; Rs arising at right angles from radius; m_1_ cell approximately triangular, petiole of m_1_ cell long, as long as m_1_ cell. Halter with stem brownish-yellow, knob grey (Fig. 1C).

Abdomen (Fig. 1A, B). Mainly yellow and black. Tergites yellow with a wide black middle stripe; first sternite pale yellow, others black. Setae on abdomen pale yellow.

**Figure 2.**
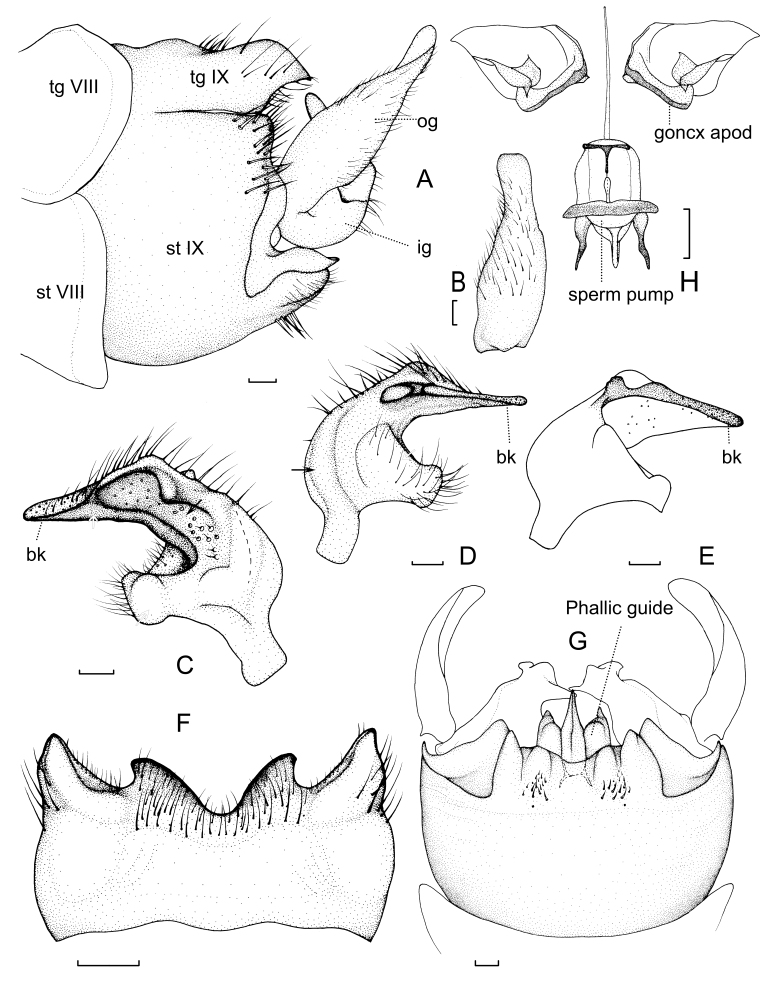
*Angarotipulatristis*, sp. n., male holotype. (A) Hypopygium, lateral view; (B) Outer gonostylus, lateral external view; (C) Inner gonostylus, lateral view; (D) Inner gonostylus, medial view; (E) Inner gonostylus, ventral medial view; (F) Tergite nine, dorsal view; (G) Hypopygium, ventral view; (H) Gonocoxal apodeme andsperm pump, dorsal view. Scale bar (A–G): = 0.2 mm, Scale bar (H): = 0.25 mm. For abbreviations, see material and methods. Black arrows: depressions; Hollow arrows: protrusions.

Hypopygium (Fig. 2A–G). Gonoxite separated from sternite IX by suture, reduced in size, slightly extended, posterior margin sharp. Tergite IX brownish-yellow, in shape of transverse plate with two pairs of horned lobes, inner pair flat and darkened along margin, covered with setae; outer pair stout, narrowed towards apex, dark brown with black apex, inner and lateral margins with less setae (Fig. 2F); posterior margin of sternite IX with two tufts at middle. Phallic guide with a pair of petal-shaped projections on both sides (Fig. 2G). Outer gonostylus yellow, fleshy and stout, apical third narrower, longer than broad (Fig. 2A, B). Inner gonostylus with an irregular depression, inside of depression with short setae and distinct trichopores; beak dark brown, apex raised edge, with transparent trichopores and lots of dorsal setae, inner margin of beak with narrowed raised edge; inner base of inner gonostylus with an oval elevation (Fig. 2C); in dorsal surface, beak with an elongate ridge with a concavity behind (Fig. 2D, E); posterior margin of inner gonostylus with a banded depression, inner margin with a hemispherical hairy elevation (Fig. 2D). Gonocoxal apodeme discontinuous, compressor apodeme and posterior immovable of sperm pump black (Fig. 2H).

#### Diagnosis

Body blackish, partial yellow on the side of the abdomen; Rs arising at right angles from radius; m_1_ cell approximately triangular; posterior margin of tergite IX with two pairs of horned lobes; outer gonostylus apical third narrower; beak dorsally with an elongate ridge with a concavity behind.

#### Etymology

The name of this species is derived from the Latin word meaning "dull-coloured," referring to the colour of the blackish body.

#### Distribution

China (Sichuan).

#### Remarks

This new species is similar to *A.frommeri* (Alexander, 1966), based on the shape of hypopygium. However, in *A.frommeri*, the body is greyish-yellow; Rs arises at an acute angle from R.; the outer gonostylus has the lateral projections; and tergite IX has no projection (Alexander 1966). The body colour of new species is similar to *A.qinghaiensis* Yang and Yang, 1996 from Qinghai, China. However, in *A.qinghaiensis*, the abdomen is completely black, the posterior margin of tergite IX is blunt without projection (Yang and Yang 1996).

### Angarotipula laetipennis

(Alexander, 1935)

E956E59B-0519-5349-95EA-CBB26BE283E6

#### Materials

- **Type status:** Other material. **Occurrence:** recordedBy: Yang Qi-Cheng and Yang Hao-Cheng; lifeStage: egg; larva; pupa; adult; **Taxon:** kingdom: Animalia; phylum: Arthropoda; class: Insecta; order: Diptera; family: Tipulidae; genus: Angarotipula; specificEpithet: *laetipennis*; scientificNameAuthorship: (Alexander, 1935); taxonomicStatus: accepted; **Location:** continent: Asia; country: China; countryCode: CN; stateProvince: Yunnan; county: Honghe Hani and Yi Autonomous Prefecture; municipality: Luxi; verbatimElevation: 1710 m; verbatimLatitude: 24°33'N; verbatimLongitude: 103°45'E; **Identification:** identifiedBy: Yang Qi-Cheng; **Event:** startDayOfYear: 12; endDayOfYear: 145; year: 2020; month: 5; day: 1; habitat: Rice field; lotus pond; eventRemarks: Angarotipulalaetipennis were reared at the outbreak of the COVID-19 epidemic in China, 2020.; **Record Level:** type: PhysicalObject
- **Type status:** Other material. **Occurrence:** recordedBy: Yang Qi-Cheng; lifeStage: egg; larva; pupa; adult; **Taxon:** kingdom: Animalia; phylum: Arthropoda; class: Insecta; order: Diptera; family: Tipulidae; genus: Angarotipula; specificEpithet: *laetipennis*; scientificNameAuthorship: (Alexander, 1935); taxonomicStatus: accepted; **Location:** continent: Asia; country: China; countryCode: CN; stateProvince: Yunnan; county: Honghe Hani and Yi Autonomous Prefecture; municipality: Luxi; locality: Xiaoxilong (小习龙), Wujiepu town.; verbatimElevation: 1700 m; verbatimLatitude: 24°32'N; verbatimLongitude: 103°34'E; **Identification:** identifiedBy: Yang Qi-Cheng; **Event:** startDayOfYear: 119; endDayOfYear: 145; year: 2020; month: 4; day: 29; habitat: Rice field (Fig. 11A); lotus pond (Fig. 11B); eventRemarks: Angarotipulalaetipennis were reared at the outbreak of the COVID-19 epidemic in China, 2020.; **Record Level:** type: PhysicalObject

#### Description

**Figure 3.**
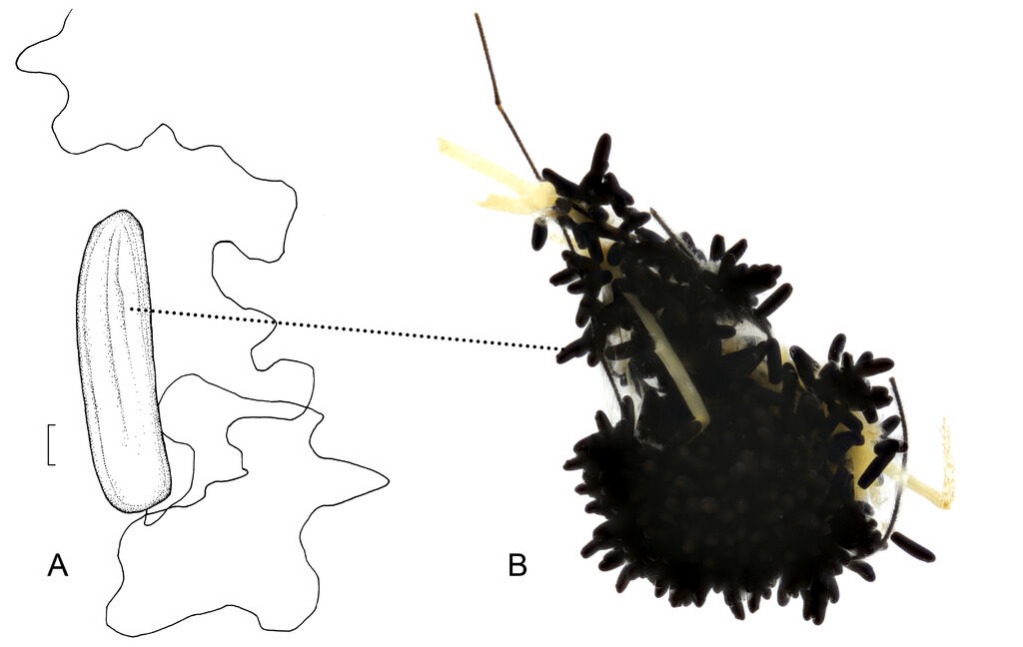
Eggs of *Angarotipulalaetipennis*. **A** egg, ventral view; **B** egg mass. Scale bar: = 0.1 mm.

**Eggs** (n > 50, produced by the same female): length 0.7–0.75 mm, middle width 0.2 mm. Black, cylindrical; apical side with a round micropyle, ventral surface with slightly longitudinal indentations. Basal side with a long terminal filament, which is more than 6 times the length of egg (Fig. 3A).

**Figure 4.**
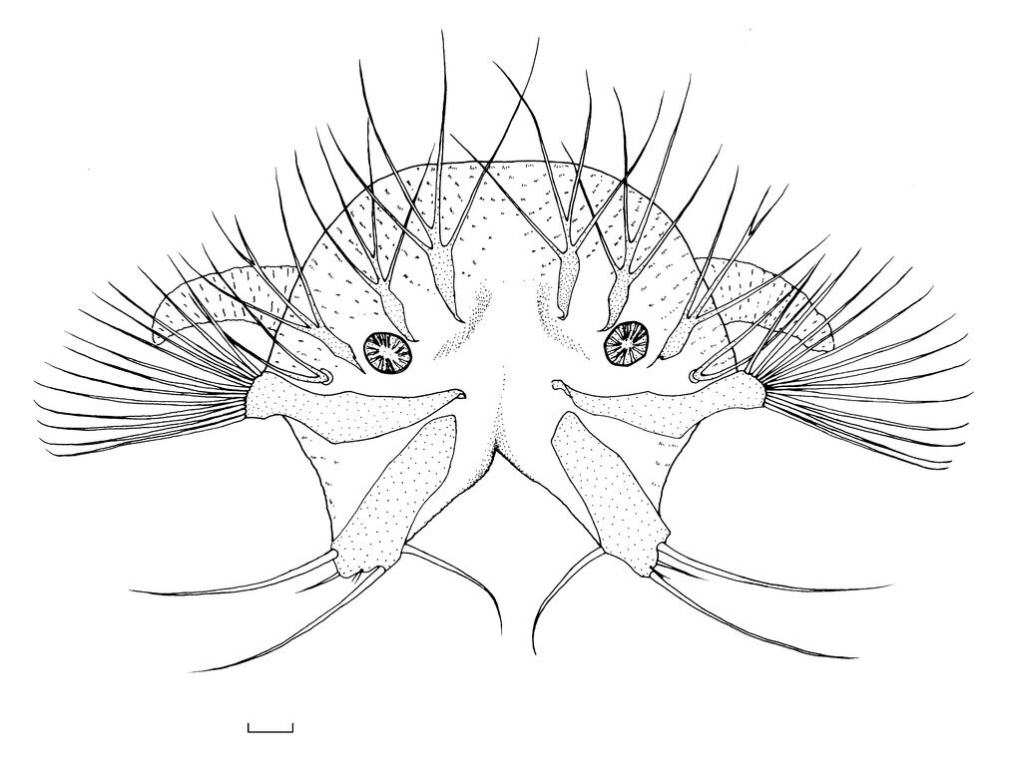
Spiracular disc of first instar of *Angarotipulalaetipennis*, dorsal view. Scale bar = 0.05 mm.

**Larvae**:

**1st instar** (n = 7, produced by the same female). Length 2.0–2.2 mm, width 0.35–0.4 mm, spiracular disc width 0.5–0.55 mm. Body cream coloured and partially translucent, body surface with pale brown corrugated pubescence, dorsal pubescence darker than those on ventral surface. Head capsule brown, posterior margin black. Spiracular disc white, with eight fusiform structures, the end structures are smaller, each of which bear moderately bifurcated long bristles; innermost pair of fusiform structures with three to five (usually four) bristles; laterally from it, the pair with three to five (usually four) bristles; outermost pair on outside of spiracles with three bristles; locations of bifurcations of these structures not fixed and the number of bifurcations not absolutely symmetrical. Spiracle small, with pale brown margin, distance beween spiracles 4 times as long as width of spiracle. Lateral lobe below spiracle, surface sclerotised, roughly sickle-shaped, tip rounded, bearing twelve to thirteen long bristles; ventral lobe finger-like with three long stiff bristles, a short stiff bristle and two tiny bristles, close together. Ventral surface of anal segment with two pairs of long anal papillae and anal papillae white, translucent (Fig. 4).

**Figure 5.**
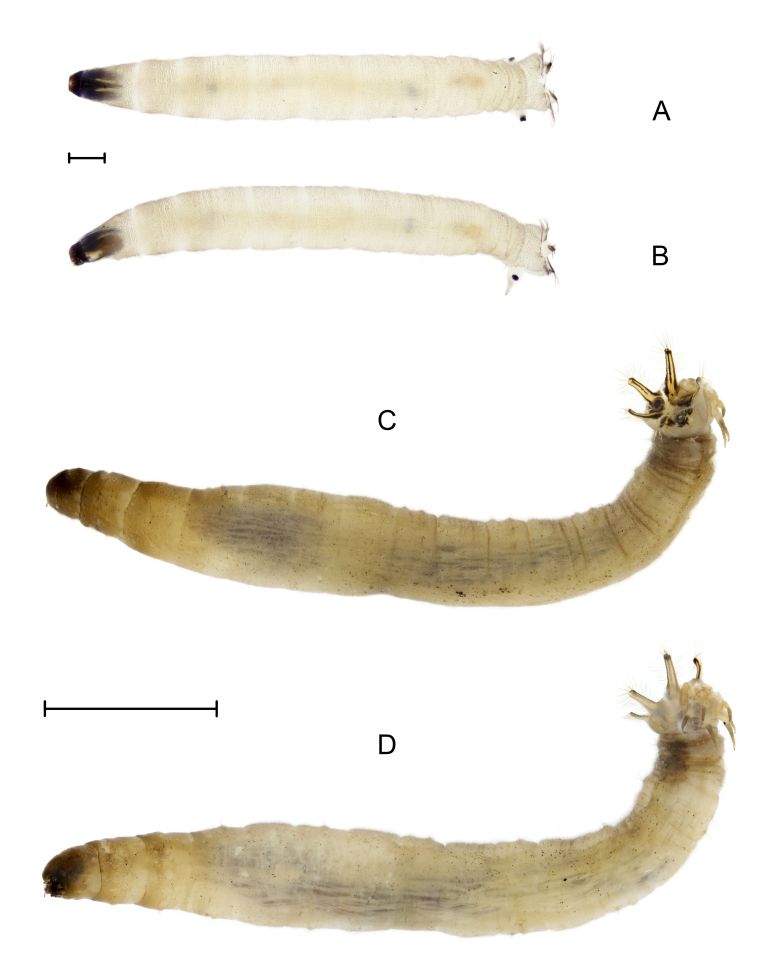
*Angarotipulalaetipennis*, larvae. **A, B** second instar, dorsal and lateral view; **C, D** last instar, dorsal and lateral view. Scale bar: A, B = 0.5 mm; C, D = 5 mm.

**2nd instar** (n = 4): length 3.0–4.0 mm, width 0.4–0.5 mm, spiracular disc width 0.65 mm. Morphological characteristics are similar to 1st instar, but head capsule darker than 1st instar; inside pair of ventral anal papillae longer than those of 1st instar and tips of outside pair of ventral anal papillae blunt and black (Fig. 5A and B).

**Figure 6.**
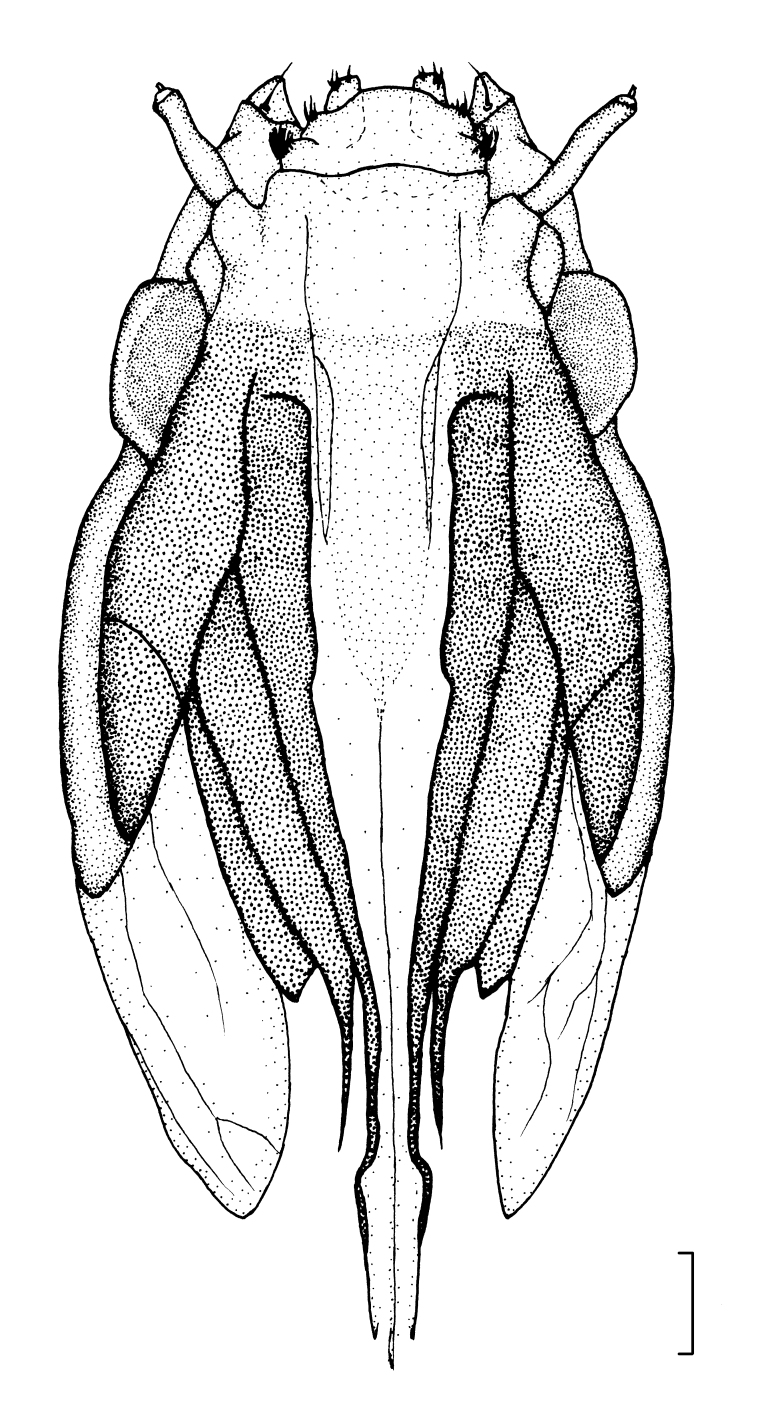
*Angarotipulalaetipennis*, head capsule of last instar, dorsal view. Scale bar = 0.2 mm.

**Figure 7.**
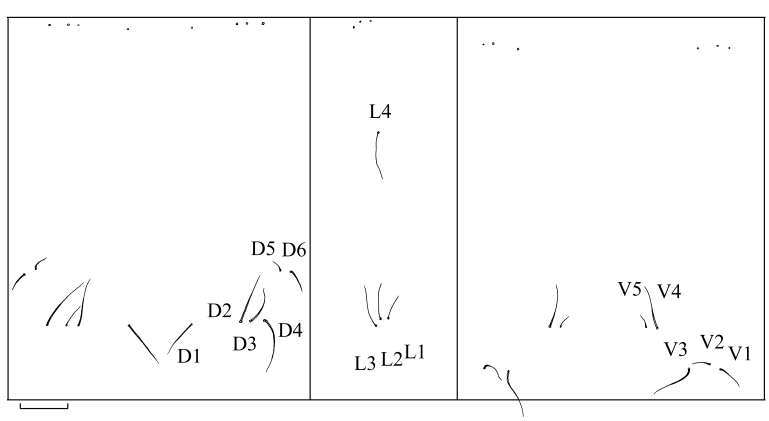
*Angarotipulalaetipennis*, last instar, dorsal, lateral and ventral chaetotaxy of abdominal segment. Scale bar = 0.5 mm.

**Male last instar** (n = 3): length 15.0–24.0 mm, width 2.0–3.0 mm, spiracular disc width 2.2–3.0 mm. **Female last instar** (n = 4): length 22.0–28.0 mm, width 2.5–3.8 mm, spiracular disc width 2.8–4.0 mm. Body brown and partially translucent, body surface with brown corrugated pubescence. Head capsule length 2.4 mm, width 1.2 mm, black, strongly sclerotised medially. Incision extending to 1/4 length of head, ends of internolateralia with a pair of long spiny processes and a pair of small triangular processes; internolateralia longer than externolateralia. Both labrum and clypeus with two pairs of brush-like setae, the smaller one on inside and the larger one on outside. Antenna cylindrical (Fig. 6). Posterior sections of abdominal segments II–VII with thin brown macrosetae arranged as follows: dorsal setae D4 longest; D5 and D6 shorter, above D3 and D4; pleura with L1, L2 and L3 close together, at level between two rows of dorsal macrosetae, L4 longest, above L1, L2 and L3; ventral setae V1, V2 and V3 close together and positioned laterally, V4 and V5 medial and anterior to latter, V3 and V4 longer （Fig. 7）. Spiracular disc with six long spiracular lobes, three times as long as width at base, ventral lobes longest; lobes fringed with long setae; thin black longitudinal lines extending along entire length of each lobe and sclerotised triangular areas at base, line of dorsal lobe thinnest, line of ventral lobe thickest. Distance between spiracles longer than width of spiracle. Three pairs of long anal papillae and spiracular lobes similar in size (Fig. 5 C, D).

**Other instars** similar to last instar. Colour cream to brown.

**Figure 8.**
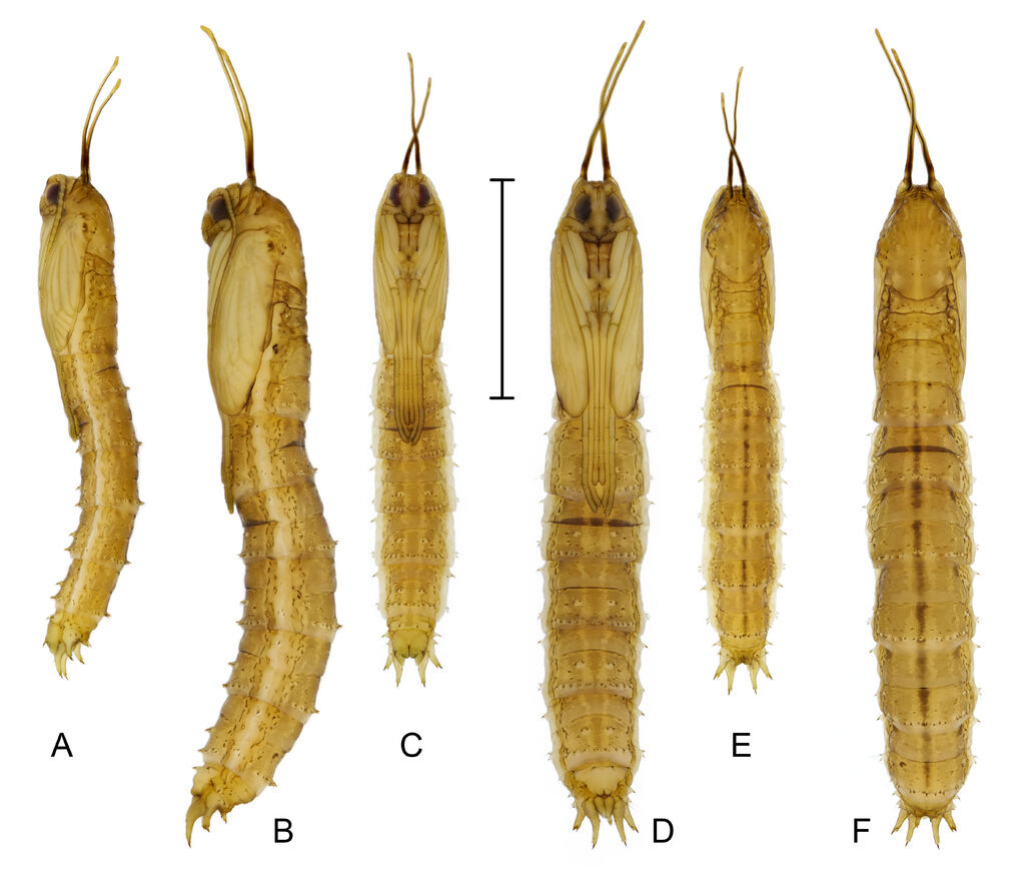
*Angarotipulalaetipennis*, pupae, (A) (C) (E) male, lateral, ventral and dorsal view; (B) (D) (F) female, lateral, ventral and dorsal view; Scale bar: = 1.0 mm.

**Pupae** (n = 9):

**Male** (Fig. 8A, C and E). Length 14.0–22.0 mm, width 1.7–2.2 mm. Pupa glabrous, yellowish-brown with faint dark brown median longitudinal line dorsally, yellow on both sides of longitudinal line; lateral sides somewhat flattened, pale yellow. Two mesonotal respiratory organs long, slightly different lengths, about 1/4 length of body, tips hammer-shaped, outer margin with groove-shaped spiracle. Mesothorax with three pairs of setae close together and located laterally; sheath of halter covered by wing sheath. Tarsal sheaths of mesothoracic and metathoracic legs curved inwards, longer than those of prothoracic legs. Abdominal segments divided into anterior and posterior sections, segments II–VII with following arrangement of spines: 11–14 tiny spines along posterodorsal edges, 1–2 spines anterolaterally; lateral parts with large spines anteriorly and three smaller spines posteriorly; posteroventral section, two small spines on either side of mid-line and row of 12–14 small spines near posterior edge. Many spines with fine setae near base. Segments VIII and IX narrower; segment IX with three pairs of sheaths over larval spiracular lobes; dorsal sheath shortest, tips of sheaths with two straight spines; lateral and ventral sheaths long, curved, tips sharp, with small spines variable in number, gonostyli sheaths with two spines ventrally.

**Female** (Fig. 8B, D and F). Length 18.0–26.0 mm, width 2.0–2.8 mm. Similar to male, except for terminal segments. Cercal sheath short, extending nearly half length of ventral spiracular sheath; hypogynial sheath short, shallowly lobed, bluntly tipped, anterior margin with four spines, many spines with a seta near base.

#### Diagnosis

**Larvae**

Incision of head capsule extending to 1/4 of head, with a pair of long spiny processes and a pair of small triangular processes; internolateralia longer than externolateralia.

**Pupae**

Two mesonotal respiratory organs nearly long, about 1/4 length of body.

**Adult**

Posterolateral margin of tergite IX with a pair of spine-like processes; inner gonostylus with a small spine-like process ventrally (Alexander 1935).

#### Distribution

China (Fujian, Guizhou, Shaanxi, Sichuan, Yunnan).

#### Biology

In Luxi County, Yunnan Province, the adults of *Angarotipulalaetipennis* can be observed in late April. In Luxi County, adults are more common in rice fields and artificial ponds. Adults have strong positive phototaxis. Females are able to lay eggs on the same day after mating. Under natural conditions, the eggs are attached to other things, such as water plants to form egg masses. Females can eject the eggs quickly without choice when it is pinched by the body. Eggs mostly hatch in 2–4 days above 22°C. Larvae move after evening and feed on brown algae and chlorella. Old instar larvae gnawed the foam box and hornwort, but they did not seem to feed on live hornwort directly. The moulting time of larvae varies according to the temperature and food. Generation overlap is found. The last instar larvae pupate on any attachments near the water surface generally. Around 29°C, the pupae usually emerge at night, but a few of the pupae emerge during the day after 4–5 days. Adults mate immediately after eclosion, male adults customarily force the incompletely ossified females. Both males and females can mate for multiple times. Adults survive for only three days without water, but they could live for more than a week in the state of feeding water.

#### Remarks

Females can mate for multiple times in the artificial environment (possibly forced) and they laid eggs on the same day. The eggs were laid out of water or on a paper towel soaked in water. The terminal filament has strong elasticity, but can be pulled apart by hand, the filaments of all eggs were anchored together and tightly attached to other things. As shown in Fig. 3B, female adults gathered all the eggs on the broken legs, making them like octopus’ eggs. The eggs hatched on the 3rd day after being laid, egg shells splitting longitudinally.

**Figure 9.**
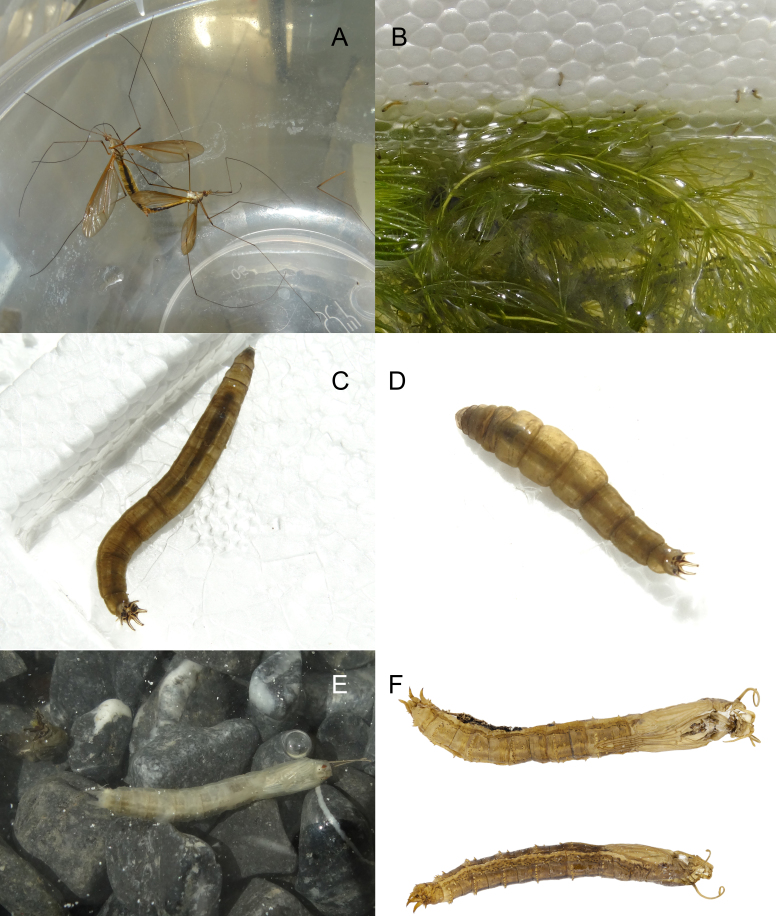
*Angarotipulalaetipennis* **A** mating; **B** young larvae foraging; **C** last instar larva; **D** last instar larva, stress state; **E** newly-pupated pupa; **F** Pupa shell, female and male.

The last instar larvae prefered to pupate near the sandy soil by water, but they did not bury their bodies in the soil. Conversely, they slightly attached to the sand and stone where they can breathe the air. Sometimes, they also pupated on the water plants close to the water. The bodies of newly-pupated pupae were white and translucent (Fig. 9E).

The last instar larvae preferred to pupate near the sandy soil by water, but they did not bury their bodies in the soil. Conversely, they slightly attached to the sand and stone where they can breathe the air. Sometimes they also pupated on the water plants closed to the water. The bodies of newly pupated pupae were white and translucent (Fig. 9E).

## Identification Keys

### Key to Chinese species (male) of genus *Angarotipula*

**Table:** 

1	Outer gonostylus with lateral process	2
–	Outer gonostylus without lateral process	4
2	Tergite 9 with a pair of spine-like process	3
–	Tergite 9 without spine-like process	*A.aspina* Liu and Yang, 2010
3	Spine-like process on posterolateral margin of tergite 9 long; inner gonostylus with a small spine in lateral view	*A.laetipennis* (Alexander,1935)
–	Spine-like process on posterior margin of tergite 9 short, inner gonostylus without spine in lateral view	*A.biprocessa* Liu and Yang, 2010
4	Tergite 9 with appendages	5
–	Tergite 9 without appendages	*A.qinghaiensis* Yang & Yang, 1996
5	Tergite 9 with claviform appendages, outer gonostylus short	*A.tumidicornis* (Lundstrom, 1907)
–	Tergite 9 with horned appendages, outer gonostylus long	6
6	Body mainly yellow, inner gonostylus with a sharp appendage posteriorly	*A.rubzovi* (Savchenko, 1961)
–	Body mainly black, inner gonostylus without sharp appendage	*A.tristis*. n

(male of *A.altivolans* (Alexander, 1935) unknown)

## Discussion

**Figure 10.**
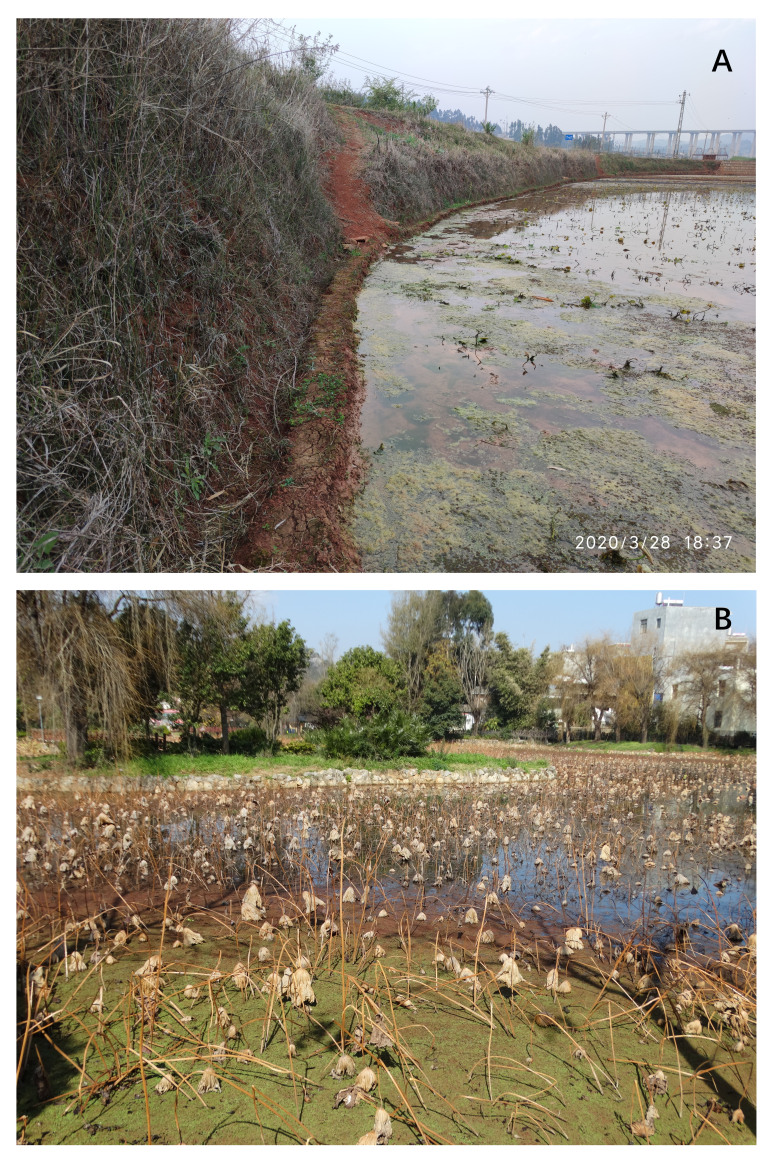
**A** Habitat of Xiaoxilong, Yunnan. Photo by Yang Qi-Cheng; **B** Habitat of Luxi, Yunan. Photo by Yang Hao-Cheng.

**Figure 11.**
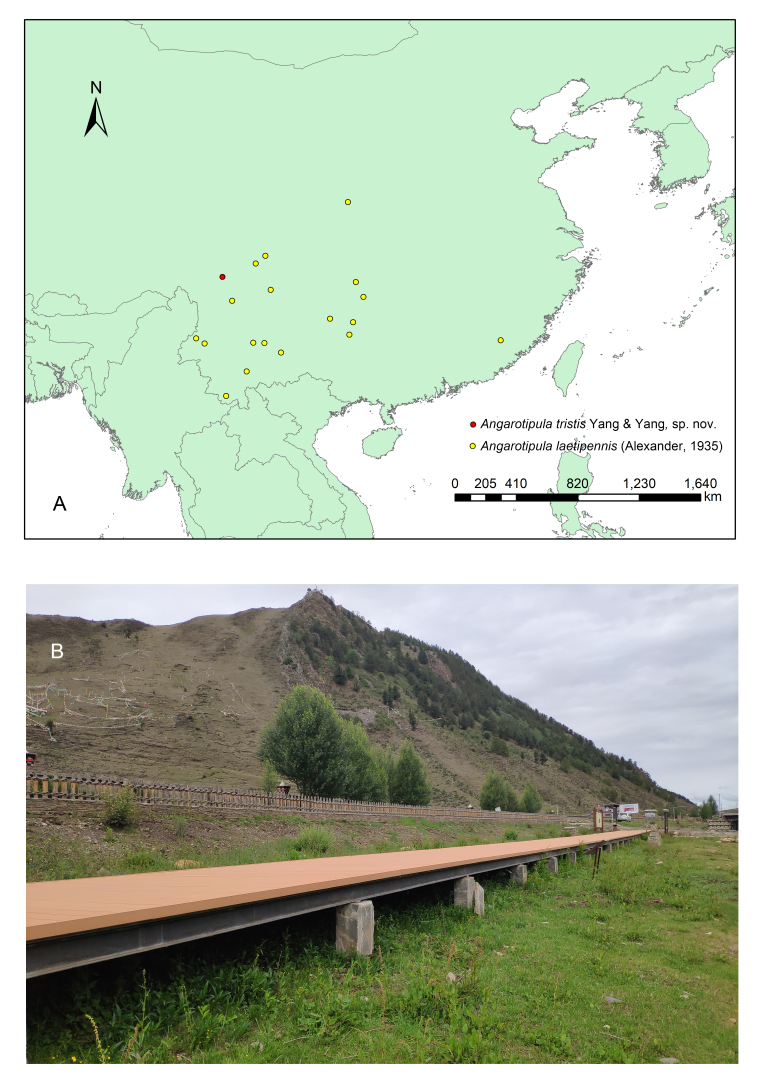
**A** Distribution map; **B** Habitat of Hongcaodi, Sichuan. Photo by Cai Xiao-Dong.

The habits of *Angarotipula* seem to be similar, they all live near freshwater ponds or swamps. The *A.laetipennis* we collected lived in paddy fields or lotus ponds (Fig. 10), while *A.tristis*, sp. n. lived in intermittent swamps (Fig. 11A). *Angarotipula* is widely distributed in the southwest of China (Fig. 11B), which may mean that Southeast Asian countries, such as Myanmar, Laos and Vietnam, also have suitable habitats. In addition, we observed the feeding habits of *A.laetipennis* for the first time and they can complete their entire life cycle by feeding on algae and have a high survival rate, which may mean that they have unique ecological niches in freshwater ponds or swamps, and we expect more studies support this view.

## Supplementary Material

XML Treatment for Angarotipula tristis

XML Treatment for Angarotipula laetipennis
